# Pancreatogastrostomy versus pancreatojejunostomy for RECOnstruction after partial PANCreatoduodenectomy (RECOPANC): study protocol of a randomized controlled trial UTN U1111-1117-9588

**DOI:** 10.1186/1745-6215-13-45

**Published:** 2012-04-27

**Authors:** Ulrich Friedrich Wellner, Sabine Brett, Thomas Bruckner, Ronald Limprecht, Inga Rossion, Christoph Seiler, Olivia Sick, Inga Wegener, Ulrich Theodor Hopt, Tobias Keck

**Affiliations:** 1 Department of General and Visceral Surgery, Hugstetter Strasse 55, 79106, Freiburg, Germany

**Keywords:** Pancreatic fistula, Pancreatoduodenectomy, Pancreatogastrostomy

## Abstract

**Background:**

Pancreatoduodenectomy is one of the most complex abdominal operations, usually performed for tumors of the periampullary region and chronic pancreatitis. Leakage of pancreatic juice from the pancreatoenteric anastomosis, called postoperative pancreatic fistula, is the most prominent postoperative complication. Retrospective studies show a significant reduction of fistula rates with pancreatogastrostomy as compared to pancreatojejunostomy, the most frequently employed method of pancreatoenterostomy. Most single-center prospective trials, however, have not validated this finding. A large multicenter trial is needed for clarification.

**Methods/design:**

RECOPANC is a prospective, randomized, controlled multicenter trial with two treatment arms, pancreatogastrostomy versus pancreatojejunostomy. The trial hypothesis is that postoperative pancreatic fistula rate is lower after pancreatogastrostomy when compared to pancreatojejunostomy. Fourteen academic centers for pancreatic surgery will participate to allocate 360 patients to the trial. The duration of the entire trial is four years including prearrangement and analyses.

**Discussion:**

Postoperative pancreatic fistula is the main reason for clinically important postoperative morbidity after pancreatoduodenectomy. The primary goal of the chosen reconstruction technique for pancreatoenteric anastomosis is to minimize postoperative fistula rate. A randomized trial performed at multiple high-volume centers for pancreatic surgery is the best opportunity to investigate one of the most crucial issues in pancreatic surgery.

**Trial registration:**

German Clinical Trials Register DRKS00000767 (2011/03/23), FSI 2011/05/31. Universal Trial Number U1111-1117-9588.

## Background

### Trial rationale

Pancreatoduodenectomy (PD) is one of the most complex abdominal operations, involving removal of the head of pancreas, duodenum and common bile duct. It is usually performed for removal of tumors of the periampullary region and for treatment of complications and pain in chronic pancreatitis. Today this operation can be performed with mortality rates below 5% at specialized centers, but perioperative morbidity remains high [1,2]. Since the beginning of pancreatic surgery [3], one of the most frequently encountered complications is leakage of pancreatic juice from the pancreatoenteric anastomosis, so-called postoperative pancreatic fistula (POPF) [4]. Depending on severity, POPF may lead to the prolongation of the hospital stay for specific treatment or even severe secondary complications. Reported rates of POPF after PD are between 20% and 30% in recent series [5,6]. The most important risk factor for development of POPF is a healthy, non-fibrotic pancreas with normal exocrine function that is found to be ‘soft’ on intraoperative palpation [7].

Many different methods of anastomosis that aim to reduce POPF rate have been described. The two main techniques practiced today are pancreatojejunostomy (PJ) and pancreatogastrostomy (PG), and for each one several subtypes have been invented [8,9].

Almost all retrospective studies comparing PG and PJ have reported a reduced POPF rate with PG, leading to a clinically important reduced POPF rate in meta-analysis [10,11]. However, three randomized controlled trials (RCTs) [12-14] did not validate this finding on meta-analysis. Only the most recent RCT of PG versus PJ showed a significant reduction in POPF rate with PG [15]; however, this trial has not yet been included in a meta-analysis. All RCTs reported so far show certain limitations; one of which is that definitions for POPF vary and are different from the currently accepted International Study Group of Pancreatic Surgery (ISGPS) definition. The only multicenter trial included in the meta-analysis showed a strong center effect and one single-center trial only included patients with a soft pancreas and small pancreatic duct. Two trials used intraoperative randomization, which may lead to a selection bias. The latest trial used a special technique of PG with gastric partitioning. In summary, generalizability of results is limited. Furthermore, the total case number in previous RCTs of around 150 provides a relatively low statistical power for detection of a difference in POPF rates.

### Aim

The objective of the RECOPANC trial is to compare the rate of POPF after PG and PJ. It is expected that POPF rates are lower with PG. There is no restriction regarding the particular technique of PG or PJ, ensuring that results may be generalized. To achieve a high case number and provide a high standard of surgical expertise, the trial is performed as a multicenter trial of specialized centers.

## Methods/design

### Trial design

RECOPANC is designed as a prospective, randomized, observer- and patient-blinded, controlled multicenter trial with two parallel study arms. The trial interventions are reconstruction of the pancreatoenterostomy by PG and by PJ, with the latter constituting the control intervention. Various techniques of PG and PJ have been described, differing in certain details (end-to-end or end-to-side PJ, with or without invagination or pancreatic duct stenting, single-layer or duct-mucosa anastomosis, technique of the associated gastro- and hepaticoenterostomy [9]). To achieve generalizable results for this trial, there is no restriction concerning the particular technique of PG or PJ.

Inclusion criteria are elective PD, age of at least 18 years and given written informed consent. Exclusion criteria are participation in another intervention-trial with interference of intervention and outcome and expected lack of compliance by the patient.

#### Recruitment and timeline

At least 14 trial centers are going to participate in the RECOPANC trial. Recruitment will be performed preoperatively at the respective trial centers by the attending physicians. The time from first patient in to last patient out is planned from June 2011 to May 2014, including two years of subject recruitment followed by one year of follow-up. The duration of the entire trial is four years including prearrangement and analysis. The actual overall duration or recruitment time may differ.

#### Randomization

To achieve comparable intervention groups, patient allocation will be concealed by preoperative randomization on the day of surgery using a centralized web-based tool (Randomizer Software, Institute for Medical Informatics, Statistics and Documentation of the Medical University of Graz, http://www.randomizer.at). Block randomization will be performed for each center to achieve equal group sizes per center. The randomization number is generated by the centralized web-based tool.

#### Blinding

At each trial center, there will be a blinded assessor who evaluates the outcome of the trial in terms of primary endpoint. The case report form (CRF) documents only whether pancreatoenterostomy was performed according to randomization. This information can be obtained from the surgeon, who cannot be blinded. Patients are kept blinded and unblinding of patients is possible if necessary for emergency treatment.

#### Primary endpoint

A primary endpoint of POPF of grade B or C has been chosen because it is usually regarded as the most important problem in pancreatic surgery. In the past, many different definitions of POPF have been used. The ISGPS introduced an international consensus definition in 2004, which includes a severity grading (grade A, B, C) [4]. As it has been shown that grade A POPF has virtually no impact on patient care and health care costs [5], we have chosen POPF grade B or C as the primary endpoint for this trial. The ISGPS definition and ‘POPF grade B or C’ have been used as an endpoint in several retrospective studies and also in current prospective randomized trials of pancreatic surgery [15,16]. Recently, detailed criteria have been published [17] as a guide to interpret the original definitions of POPF grades according to the ISGPS. These will be adhered to in the current study with one modification: application of somatostatin analogues will not lead to classification as POPF grade B or C, because somatostatin analogues are applied routinely in some centers and its effect on the reduction of pancreatic fistula risk is questionable [18]. POPF of grade B or C according to the ISGPS definition constitutes a dichotomous (yes or no) endpoint. POPF is present by definition if, on or after postoperative day 3, total amylase activity in the peritoneal drain secretion exceeds the upper normal limit of serum amylase activity threefold. POPF is of grade A when grade B or C criteria are not fulfilled. The scorers of each study center are trained during the trial initiation visit by the monitors; extensive monitoring during the trial will ensure the reliability of outcome assessment.

#### Secondary endpoints

Secondary endpoints are assessed to cover other important aspects of outcome after PD and thereby achieve a valid comparison between the two study arms. The secondary endpoints comprise two main fields. First, the most typical problems or complications of pancreatic surgery are included as perioperative secondary endpoints. To ensure comparability and generalizability of results, existent ISGPS definitions are employed here, too.

Second, long-term outcomes regarding pancreatic function and quality of life are considered. We chose 30 days, 6 months and 12 months as time points for long-term outcome evaluation visits, to ensure inclusion of oncological patients in the follow-up, where survival time is limited. Quality of life is measured by use of the well-established European Organisation for Research and Treatment of Cancer (EORTC) Quality of Life Questionnaire (QLQ)-C30 and QLQ-PAN26 (for pancreatic cancer) (EORTC Study Group on Quality of Life, Brussels, Belgium). Long-term pancreatic function is an often neglected aspect in surgical technical studies. In this trial, we assess pancreatic function by a questionnaire (included in the CRF) regarding the necessity of antidiabetic medication, pancreatic enzyme supplementation and symptoms of exocrine pancreatic insufficiency. More objective assessment of pancreatic function (oral glucose tolerance test, pancreatic secretion samplings or stool enzyme activity measurements) is not planned to date. According to our own experience, it cannot be expected that these time-consuming and expensive analyses will be performed in the majority of patients enrolled in the trial at various centers in our multicenter trial.

Definitions of secondary endpoints are:

overall mortality: death due to any cause at any time during follow-up period

POPF, grade A (ISGPS definition [4], yes or no)

delayed gastric emptying of grade B or C (ISGPS definition [19], yes or no)

postpancreatectomy hemorrhage (ISGPS definition [20], yes or no)

intra-abdominal fluid collection or abscess requiring invasive treatment (yes or no)

relaparotomy (yes or no)

necessity of completion total pancreatectomy (yes or no)

anastomotic leak other than POPF (yes or no)

wound infection requiring invasive treatment (yes or no)

septic shock: sepsis requiring catecholamine treatment (yes or no)

respiratory failure requiring invasive mechanical ventilation (yes or no)

deep venous thrombosis (yes or no)

lung embolism (yes or no)

myocardial infarction (yes or no)

stroke (yes or no)

operation time from incision to end of skin closure (minutes)

postoperative hospital stay until discharge (days)

learning curve effects

pancreatic endocrine and exocrine function (steatorrhea, necessity of oral enzyme replacement, necessity of antidiabetic or insulin therapy)

quality of life (EORTC QLQ-C30 and QLQ-PAN26, according to the scoring manual published by the EORTC Quality of Life group [21])

#### Trial locations

To achieve the highest standard of care and avoid center bias, only high-volume academic centers for pancreatic surgery are selected to participate in the trial. Minimum requirements for inclusion are at least five pancreatic head resections with each of the two reconstruction techniques (PG and PJ) per year or previous experience with at least 25 pancreatoduodenectomies of each technique. Participating centers are: Klinik für Allgemein-, Viszeral- und Transplantationschirurgie Universitätsklinikum Aachen; Klinik für Allgemein-, Viszeral- und Transplantationschirurgie Charité Campus Virchow; St. Josefs-Hospital Bochum; Klinik und Poliklinik für Allgemein-, Viszeral-, Thorax- und Gefäßchirurgie Universitätsklinikum Bonn; Klinik für Allgemein- und Viszeralchirurgie Universitätsklinikum Freiburg; Klinik für Allgemein-, Viszeral-, Thorax-, Transplantations- und Kinderchirurgie Universitätsklinikum Giessen; Klinik und Poliklinik für Allgemein-, Viszeral- und Thoraxchirurgie Universitätsklinikum Hamburg-Eppendorf; Klinik für Allgemein-, Viszeral- und Transplantationschirurgie Universitätsklinikum Heidelberg; Chirurgische Klinik Universitätsklinikum Mannheim; Klinik für Viszeral- Thorax- und Gefäßchirurgie Universitätsklinikum Marburg; Chirurgische Klinik und Poliklinik Universitätsklinikum München LMU; Klinik für Viszeral- Thorax- und Gefäßchirurgie Klinikum Rechts der Isar München; Allgemein- und Viszeralchirurgie Krankenhaus Barmherzige Brüder Regensburg; and Klinik und Poliklinik für Chirurgie Universitätsklinikum Regensburg. Further centers may join the trial.

### Statistical methods

#### Sample size calculation

**Figure 1:**
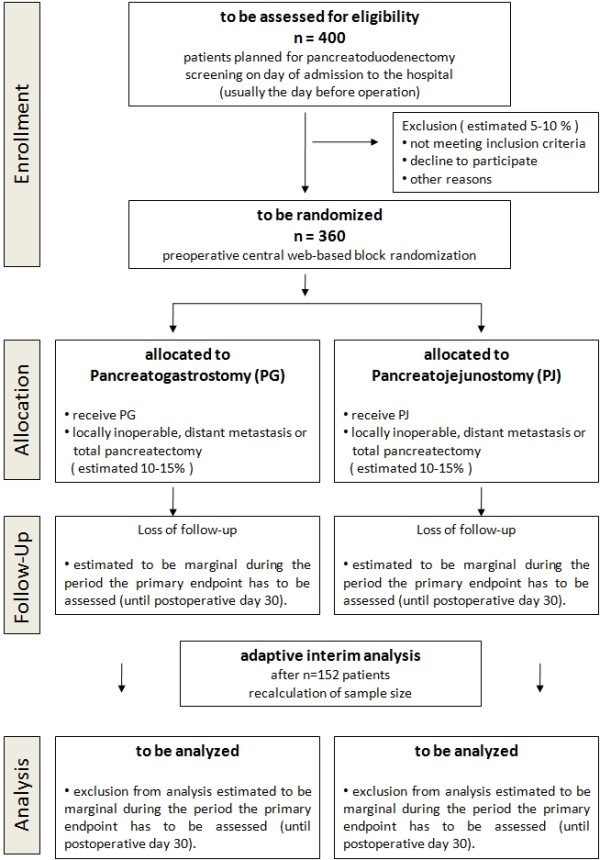
Trial flow chart.

The prior assumption is an overall rate of pancreatic fistula type B or C within 30 days after surgery in the PG group of 6% (5/82 patients) versus 16% (14/84 patients) in the PJ group. The sample size calculation is based on the primary outcome - POPF grade B or C, as reported in the randomized trials by Fernandez-Cruz *et al*. [15] and Wellner *et al*. [22]. With alpha = 5% and beta = 20%, a sample size of n = 153 per group is necessary to detect a difference between the intervention groups when applying the Chi-square test (two-sided analysis, SAS 9.1 software, SAS, Cary, NC, USA). It can be expected that including covariates of prognostic importance [7] in the logistic regression model applied in confirmatory analysis will increase the power as compared to the Chi-square test [23]. To address the uncertainty in the estimation of the rates of pancreatic fistula in the two intervention arms, an adaptive interim analysis is planned according to Bauer and Köhne [24] after recruitment of half of the necessary participants (n = 152). Assuming a drop-out rate of 15%, another 54 patients have to be randomized for a total of n = 360 patients to be allocated to this trial. It is estimated that around 400 patients will have to be assessed for eligibility. Loss to follow-up is expected to be marginal: during the period that the primary endpoint (POPF grade B or C) has to be assessed (until postoperative day 30), all patients are under continuous in-hospital observation in the perioperative period, followed by in-hospital rehabilitation for the great majority and close ambulatory observation in every case. Previous multicenter trials in pancreatic surgery support this assumption [13,16]. For the same reason, the number of subjects to be excluded from final analysis of the primary endpoint up to postoperative day 30 is estimated to be marginal. If missing values occur, they will be replaced by the imputed case analysis incorporating available reasons for missing data method described by Higgins *et al*. [25]. The resulting trial flow chart is shown in Figure 1. 

#### Analysis

The primary hypothesis is that the rate of clinically relevant POPF (Grade B or C) is lower with PG than PJ in patients undergoing partial PD. The analysis will be performed according to the standard (non-modified) intention to treat principle [26,27]. A multivariable logistic regression model will be applied for the intervention comparison of the POPF rates adjusting for age, center, surgeons experience and texture of pancreatic tissue. To address the differences already seen in the rates of POPF, an interim analysis will be conducted after recruitment of half of the necessary case numbers according to the design described by Bauer and Köhne [24]. Patients with a soft pancreas have a higher risk of developing POPF and constitute a high-risk subpopulation. These patients are expected to comprise about half of all cases [7]. If the POPF rate after PG or PJ is different in this subgroup, it will be evaluated by an exploratory subgroup analysis including statistical testing for interaction with treatment [23]. SAS software version 9.1 or higher will be used for the statistical analyses.

#### Withdrawals

Patients are free to leave the trial at any time and without giving reasons for their decision. Patients may be withdrawn from the trial at their own request; if, in the investigator’s opinion, continuation of the trial may be detrimental to the patient’s well-being; or if a PD was not performed (because of technical unresectability, metastatic disease or other reasons). The investigator decides on withdrawal of subjects from the clinical trial in the case of the latter criteria. In all cases, the reason for withdrawal must be recorded in the CRF and in the patient’s medical records. The patient must be followed up as far as possible; all examinations scheduled for the final trial day must be performed on all patients and documented. To do so the consent of the patient is necessary and will be requested.

#### Stopping rules

As described in detail by Wassmer [28], in a two-stage Bauer-Köhne design [24] addressing a two-sided problem, it is valid to perform two one-sided tests to the level α/2. The critical values for the smaller of the two one-sided *P*-values for the primary endpoint in stage one are α1 = 0.0038. Where *P >*0.0038, the trial will be continued and the sample size will be recalculated. The recalculated sample size will be furnished to the independent Data Safety Monitoring Board (DSMB) with the interim report. This board will give advice whether and how additional recruitment of patients will be feasible.

#### Premature closure

The trial may be prematurely closed by the principal investigator in consultation with the steering committee and the responsible biometrician. If termination of the trial becomes necessary, the RECOPANC steering committee will discuss this issue with the independent DSMB. Reasons that may necessitate termination of the trial include the rate or severity of serious adverse events or morbidity in the trial indicating a potential health hazard caused by the study treatment; patients’ enrolment is unsatisfactory with respect to quality or quantity; data recording is severely inaccurate or incomplete; or external evidence demands a termination of the trial.

### Administration

#### Funding

The trial will be financed by funding from the Deutsche Forschungsgemeinschaft (DFG; German Research Foundation/Project number HO810/3-1).

#### Monitoring

Clinical monitoring will be performed by the Studienzentrum Freiburg, an institution which is independent from other trial staff. Monitoring procedures will be adapted to the study-specific risks for patients, interpretation of the International Conference on Harmonisation of Technical Requirements for Registration of Pharmaceuticals for Human Use-Good Clinical Practice (GCP) E6 guidelines [29] and standard operating procedures of the Studienzentrum Freiburg to ensure patient safety and integrity of the clinical data, for example, primary endpoint in adherence to the study protocol. Monitor visits are taking place before, during and after the study. Prior to study start, all participating centers will be personally trained and introduced to all study-specific procedures during individual on-site initiation visits. Regular on-site monitoring visits are planned at all sites depending on the recruitment rate and quality of the data.

#### Data management

Central data collection and maintenance is performed by the Institute of Medical Biometry and Informatics (IMBI), Heidelberg, Germany. All protocol-required information for the clinical data collected during the trial must be entered by the investigator, or a designated representative, in the electronic CRF. Macro™ electronic data capture software (InferMed, London, UK [30]) will be used for electronic CRF coding and data management. The completeness, validity and plausibility of the data (clinical and quality of life data) are examined by validating programs, which thereby generate queries. The investigator or the designated representatives are obliged to clarify or explain the queries. If no further corrections are to be made in the database it will be declared closed and used for statistical analysis.

### Ethical considerations

#### Risks

There are no apparent risks for patients taking part in the study as they receive the usual, standard treatments and standard diagnostic assessment.

#### Informed consent

Patients will be enrolled into the study only after comprehensive information has been explained to them in an understandable way by the responsible investigator, concerning the nature, scope and possible consequences of the clinical trial. Written informed consent for the study will be obtained from each patient before any study-specific procedure and randomization is executed.

#### Safety and serious adverse events

Analysis of safety-related data is performed with respect to the frequency of serious adverse events in both treatment groups, the frequency of serious adverse events stratified by causality and the frequency of morbidity in both treatment groups. All serious adverse events that occur during the trial - from randomization to the regular end of the trial at 12 months follow-up or until premature withdrawal of the patient - must be documented on a Serious Adverse Event Form available in the investigator site file. Serious adverse events have to be reported by the attending physician to the Clinical Trials Unit Freiburg within five days of the serious adverse event becoming known.

#### Data safety monitoring board

An independent DSMB is established, consisting of two academic surgeons and a biometrician. In case of any irregularities, for example concerning the frequency or type of serious adverse event reported, the principal investigator will inform the members of the independent DSMB without delay. At least once every 12 months, the DSMB will receive a written safety report. The members of the DSMB then report the result of the benefit and risk assessment to the principal investigator and will give recommendations concerning the continuation of the trial.

#### Approval

Before the start of the trial, the trial protocol, informed consent document, and any other appropriate documents will be submitted to an independent ethics committee. The protocol has been approved by the ethics committee of the medical faculty Freiburg and must be approved by the ethics committees of all other participating centers before patient recruitment is started. All amendments will also be submitted to the independent ethics committees.

#### Good clinical practice

The procedures set out in this trial protocol, pertaining to the conduct, evaluation and documentation of this trial, are designed to ensure that all persons involved in the trial abide by GCP and the ethical principles described in the current revision of the Declaration of Helsinki [31]. The trial will be carried out in accordance with local legal and regulatory requirements.

#### Registration

The RECOPANC trial has been assigned a universal trial number (UTN: U1111-1117-9588) and registered in the German Clinical Trials Register (DRKS-ID: DRKS00000767) as of 23 March 2011. The first patient was randomized on 31 May 2011.

## Discussion

About one hundred years after the first successful PD was performed by Walther C.E. Kausch [3], this complex operation can be performed routinely and with low mortality at centers for pancreatic surgery. One of the main problems contributing to a high postoperative morbidity remains the occurrence of POPF.

Three of four randomized trials have not supported the conclusion of numerous retrospective studies suggesting a reduced POPF rate with PG when compared to PJ. The RECOPANC trial is designed to show a significant reduction of POPF with PG. Noteworthy features in comparison to previous trials include a large sample size and adaptive interim analysis for recalculation of sample size, ensuring high statistical power; planned analysis of the high-risk subgroup of patients with a soft pancreas; no restriction in terms of sub-techniques of PG and PJ and a multicenter approach, leading to generalizable results; use of the ISGPS definitions for primary and secondary endpoints, which makes results comparable to other studies; and inclusion of high-volume academic centers only, ensuring highest standard of care.

It is our aim that this trial will help to minimize postoperative complication rates of PD.

## Trial status

At the time of manuscript submission, the RECOPANC trial is recruiting patients. The first subject was randomized on 31 May 2011.

## Abbreviations

CRF: Case report form; DFG: German Research Foundation; DSMB: Data Safety Monitoring Board; EORTC: European Organisation for Research and Treatment of Cancer; GCP: Good Clinical Practice; IMBI: Institute of Medical Biometry and Informatics, Heidelberg; ISGPS: International Study Group of Pancreatic Surgery; PAN26: Specific QLQ to supplement the QLQ-C30 in Patients with pancreatic cancer; PD: Pancreatoduodenectomy; PG: Pancreatogastrostomy; PJ: Pancreatojejunostomy; POPF: Postoperative pancreatic fistula; RCT: Randomized controlled trial; QLQ: Quality of life questionnaire; QLQ-C30: Core cancer QLQ of the EORTC.

## Competing interests

The authors declare that they have no competing interests.

## Authors’ contributions

UFW writing of the manuscript, establishment of the trial protocol. SB trial monitor, establishment of the trial protocol. TB trial biometrician, statistical methods, establishment of the trial protocol. RL data management at the IMBI, establishment of the trial protocol. IR coordination of trial conduct, establishment of the trial protocol. CS project management at the Study Center of the German Surgical Society, establishment of the trial protocol. OS coordination of trial conduct, establishment of the trial protocol. IW coordination of trial conduct, establishment of the trial protocol. UTH principal investigator, establishment of the trial protocol. TK trial coordinator, establishment of the trial protocol, finalization of the manuscript. All authors read and approved the final manuscript.
